# Role of red blood cells in clinically relevant bleeding tendencies and complications

**DOI:** 10.1016/j.jtha.2023.05.009

**Published:** 2023-05-18

**Authors:** Riitta Lassila, John W. Weisel

**Affiliations:** 1 Research Program Unit in Systems Oncology, Oncosys, Medical Faculty, University of Helsinki, Helsinki, Finland; 2 Coagulation Disorders Unit, Department of Hematology, Helsinki University Hospital, Helsinki, Finland; 3 Department of Cell and Developmental Biology, School of Medicine, University of Pennsylvania, Philadelphia, Pennsylvania, USA

**Keywords:** anemia, bleeding disorders, hemostasis, red blood cells, von Willebrand disease

## Abstract

The multiple roles of red blood cells (RBCs) are often neglected as contributors in hemostasis and thrombosis. Proactive opportunities to increase RBC numbers, either acutely or subacutely in the case of iron deficiency, are critical as RBCs are the cellular elements that initiate hemostasis together with platelets and stabilize fibrin and clot structure. RBCs also possess several functional properties to assist hemostasis: releasing platelet agonists, promoting shear force–induced von Willebrand factor unfolding, procoagulant capacity, and binding to fibrin. Additionally, blood clot contraction is important to compress RBCs to form a tightly packed array of polyhedrocytes, making an impermeable seal for hemostasis. All these functions are important for patients having intrinsically poor capacity to cease bleeds (ie, hemostatic disorders) but, conversely, can also play a role in thrombosis if these RBC-mediated reactions overshoot. One acquired example of bleeding with anemia is in patients treated with anticoagulants and/or antithrombotic medication because upon initiation of these drugs, baseline anemia doubles the risk of bleeding complications and mortality. Also, anemia is a risk factor for reoccurring gastrointestinal and urogenital bleeds, pregnancy, and delivery complications. This review summarizes the clinically relevant properties and profiles of RBCs at various steps of platelet adhesion, aggregation, thrombin generation, and fibrin formation, including both structural and functional elements. Regarding patient blood management guidelines, they support minimizing transfusions, but this approach does not deal with severe inherited and acquired bleeding disorders where a poor hemostatic propensity is exacerbated by limited RBC availability, for which future guidance will be needed.

## 1 | INTRODUCTION

**FIGURE 1:**
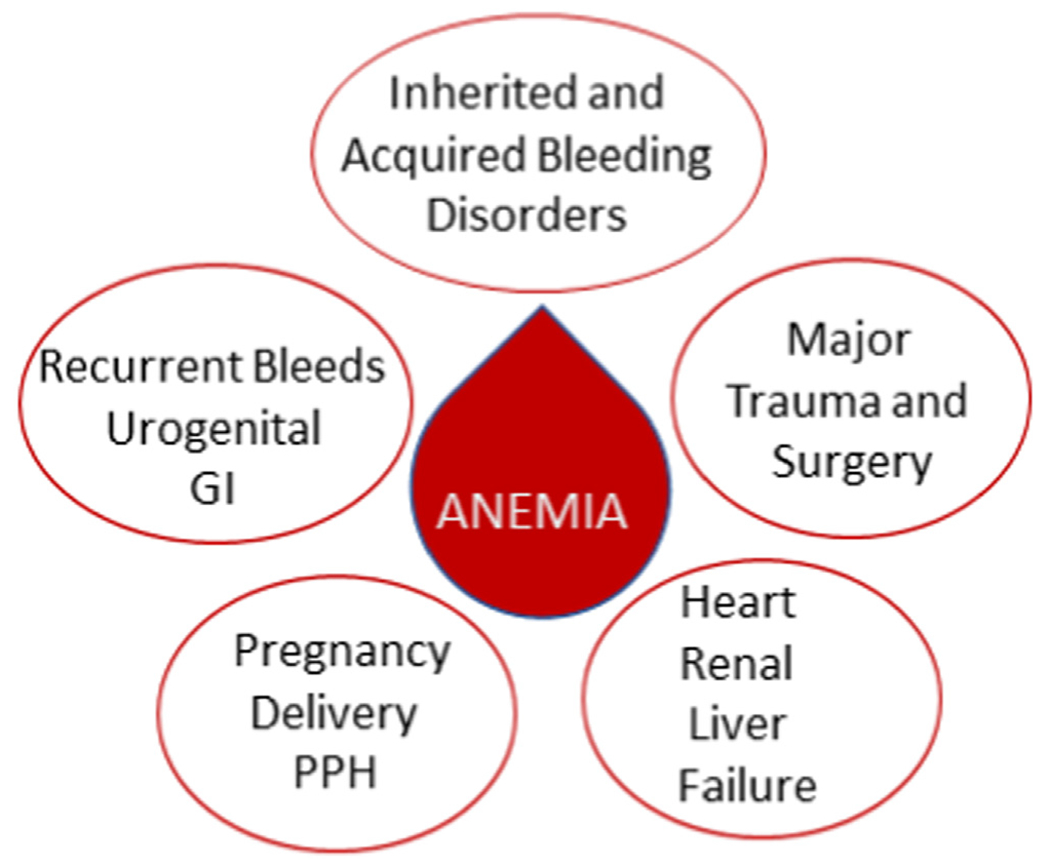
Clinical scenarios where anemia should be the focus of patient evaluation and follow-up for making management decisions. GI, gastrointestinal; PPH, postpartum hemorrhage.

The contributions of cells, plasma proteins, vascular wall, and flow dynamics are familiar building blocks for hemostasis. The numbers of red blood cells (RBCs) may be low due to deficiency in hematopoiesis (bone marrow failure), major bleeds, or cellular destruction because of membrane abnormalities (sickle cell trait or hemoglobinopathies, antibodies or malaria, or other pathogens and hemolysis associated with paroxysmal nocturnal hemoglobinuria with a thrombotic tendency). However, the most common reason for anemia is iron deficiency, either due to poor absorption of iron or loss of RBCs due to subtle bleeding, impairing oxygen delivery. All these disorders lead to coagulation abnormalities, which need special and proactive attention. Here, we will focus mostly on disorders with decreased RBC numbers from the point of view of hemorheology, bleeding tendency, and complications. After discussing the critical mechanisms of hemorheology, we will identify the most important disorders where anemia should be under active evaluation to be managed if possible (Figure 1). Finally, we will return to the bench for other valuable RBC-associated functional properties, including clot contraction and RBC microvesicles, to be further considered in clinics as well.

**FIGURE 2:**
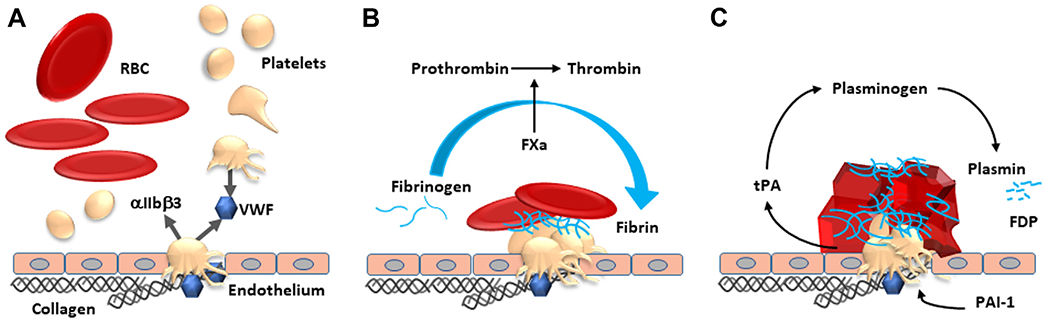
Three stages (A–C) in which red blood cells (RBCs) contribute to hemostasis. (A) Primary hemostasis and platelet activation. RBCs, by increasing local vessel wall shear forces, induce margination and activation of platelets at an injured vascular wall, unfold von Willebrand factor (VWF), and enhance platelet-vascular wall interactions. (B) Thrombin generation and fibrin formation. RBCs provide phosphatidylserine, which is a procoagulant surface to assemble coagulation factors and contribute to thrombin generation, platelet activation, and fibrin formation. (C) Fibrin stability, clot contraction, and formation of polyhedrocytes. RBCs have a procoagulant surface, enhancing thrombin generation, and bind to platelets via interactions between platelet integrin αIIbβ3 (or glycoprotein IIb/IIIa) and endothelial cell intercellular adhesion molecule 4 (ICAM-4) and to fibrin(ogen) via a β3 integrin, which aids in clot stabilization and prevents early fibrinolysis. During clot contraction, RBCs are compressed to tightly packed polyhedrocytes, forming an impermeable seal. (A) The activation and secretion of platelets, (B) clot structure from thrombin generation and fibrin polymerization, as well as activation of coagulation factor XIII, a fibrin stabilizing factor, all affect fibrinolysis together with clot contraction (C). Integrin αIIbβ3 is a receptor of VWF (high shear rates) and fibrinogen (low shear rates). FXa, activated coagulation factor X, FDP, fibrin degradation products. including D-dimer; PAI-1, plasminogen activator inhibitor; tPA, tissue plasminogen activator.

The critical role of RBCs in primary hemostasis is often inappropriately overlooked, both in the basic research of thrombosis and hemostasis and their clinical implications [1]. RBCs modify hemorheology, which involves the important mechanical force to keep platelets close to the vascular wall surface, mechanically enhancing their interaction with injury sites [2]. The resulting flow dynamics are especially strong in small arteries, the microvasculature, and stenosed large arteries, assisting platelets to cease a bleed. RBCs support not only platelets but also the unfolding of von Willebrand factor (VWF), which is a critical element to capture platelets by exposing multiple binding sites for platelets under high shear rates at sites of vascular injury [3] (Figure 2). Moreover, RBC surface phospholipids are able to assemble coagulation factors, generate thrombin, and convert fibrinogen to fibrin [4]. Finally, RBCs actively bind to both platelets and fibrin and not just get trapped in the clot [5]. Moreover, clot contraction has a significant impact on hemostasis via compression and structural alteration of the RBCs to yield tightly packed polyhedrocytes, which form an impermeable seal at the injury site [6–8] (Figure 2). This mechanism enhances clot stability (eg, during the management of factor VIII [FVIII] replacement therapy in severe hemophilia) [6]. This observation is a clear example of how the processes of primary and secondary hemostasis are integrated. As to fibrinolysis, which is beyond the scope of this review, we refer the reader to 3 recent contributions [9–11].

Hemorheology in practical terms promotes hemostatic interactions, meaning that the larger the RBC numbers, the stronger are the blood flow forces for targeting and attaching platelets onto the vascular wall. RBCs will flow in the middle of the blood vessels, assisting the margination of platelets nearest to the endothelium or vascular surfaces [1–3]. RBC quantity, vasoconstriction, and narrowed vessel wall lumen will enhance VWF to mediate platelet adhesion upon vascular wall injury [2,3]. VWF, deriving from the endothelium, plasma, and platelets, bridges platelets to collagen to initiate hemostasis and stabilize the initial platelet plug under high blood flow conditions.

In the early studies of hemorheology, it was shown that at shear rates between 50 and 2600 1/s, prevailing in veins to medium- and small-sized arteries, the highest shear rates lead to most platelet recruitment to the subendothelial surface [2]. At a level of hematocrit <30%, the enhancing physiological impact of shear forces on platelet margination and consequent adhesion to the injured vascular wall is minimized. This observation forms the basis for the value of increasing the hematocrit level with transfusion in appropriate patient cases, especially in ones with a hemostasis defect (ie, von Willebrand disease [VWD], platelet defects, and hemophilia) or in the case of strong antithrombotic medication upon a bleed.

## 2 | CLINICAL CONSIDERATIONS

### 2.1 | Inherited bleeding disorders

The clinical implications of low RBC numbers are most associated with the bleeding phenotype of deficient primary hemostasis (ie, VWF in types 1 and 2 and even in type 3 VWD) and platelet function disorders and partially observed in other rarer bleeding disorders, including hemophilia [6]. On top of any specific coagulation defect causing the bleeding disorder, anemia brings a synergistic risk factor for poor hemostasis (Figures 1 and 2). This consideration is typically relevant for acute as well as recurring bleeds of the gastrointestinal (GI) tract, hematuria, menorrhagia, and invasive interventions, including surgery. As an example, one study reported that 75% of female patients with VWD are at risk of recurrent menorrhagia and iron deficiency anemia compared with 16% of controls without VWD [12]. Paradoxically, both progressive anemia and erythrocytosis can be clear risk factors for thrombosis and even mortality [13], showing that RBC numbers matter. One feature of iron deficiency anemia is thrombocytosis, which may enhance platelet margination during vascular injury.

### 2.2 | Acquired bleeding disorders

#### 2.2.1 | Bleeding with antithrombotic medication

The most common acquired bleeding disorders are associated not only with frequently used antithrombotic medications, mostly antiplatelet agents, but also with anticoagulants, either individually or in combination. Anticoagulant medication is a frequent cause of adverse drug events in the emergency department, where bleeding complications are proliferating due to the increasing usage of antithrombotic medication and after thrombolytic therapy [14]. It may come as a surprise that if a patient with atrial fibrillation is even borderline anemic (hemoglobin <130 mg/L in men and <120 mg/L in women [World Health Organisation criteria for anemia]) when treatment with any of the oral anticoagulants is initiated, the risk of bleeding complications and mortality is doubled in comparison to that in patients who have normal erythrocyte counts [15–17].

Platelet inhibitors affecting primary hemostasis cause the same problems [18]. Therefore, the combination of antiplatelet agents and anticoagulants is justified for patients with a significant thrombotic propensity, without or with only minor risk factors for bleeding, and in that case only for a limited period. In the case of cardiovascular complications in a patient with inherited bleeding disorders, ie, VWD or hemophilia, recent expert guidance documents for managing acute thrombosis and beyond are available [19] and emphasize the management of anemia [20].

Among patients with atrial fibrillation using dabigatran or warfarin in the large The Randomized Evaluation of Long-Term Anticoagulation Therapy [RELY] trial, 12% had anemia already at the initiation of the study [15]. The anemic patients carried an elevated cardiovascular risk profile. Anemia, particularly, was associated with the risk of bleeding complications, at a safety hazard ratio (HR) exceeding 2, which resulted in cessation of the anticoagulant and in turn created a risk for systemic embolization and mortality (HR, 1.4 and 1.5, respectively). This is the case irrespective of the anticoagulant chosen, except that the site of the bleed with warfarin is more often intracranial than with direct oral anticoagulants [15–17].

In another study (The Rivaroxaban Once Daily Oral Direct Factor Xa Inhibition Compared with Vitamin K Antagonism for Prevention of Stroke and Embolism Trial in Atrial Fibrillation [ROCKET AF]) of patients with atrial fibrillation on rivaroxaban or warfarin, 5% of the patients experienced a GI bleed, which affected mostly the upper GI tract [16]. Major GI bleeds occurred more (HR, 1.4; 95% CI, 1.2-1.7) in the rivaroxaban group than in the warfarin group. The independent risk factors for major bleeds included baseline anemia, history of a GI bleed, and coadministration of aspirin.

Finally, also in case of apixaban (Apixaban for Reduction in Stroke and Other Thromboembolic Events in Atrial Fibrillation [ARISTOTLE] trial) among patients with atrial fibrillation, baseline anemia (according to World Health Organisation criteria) was associated with almost doubling (HR, 1.9) of bleeding complications and aligned with significant increased mortality after follow up at 1.8 years [17]. Again, anemia occurred in 12% of the patients at the baseline, and new anemia developed in 30% of the patients during the study. Overall, in patients with atrial fibrillation, anemia should be closely monitored during all types of anticoagulant therapy and solving the underlying causes of anemia is critical. In patients with acute coronary syndromes who are on platelet inhibitors, anemia is also clearly associated with bleeding risks [18]. Successful proactive management of anemia, most often iron deficiency anemia, should be the target to avoid severe complications in all patients, along with all types of antithrombotic treatment. Obviously, endoscopies are needed to identify and manage the tissue injuries or malignancies causing GI bleeds.

#### 2.2.2 | Major trauma and surgery

In trauma, the effects of anemia are exacerbated as a result of loss of blood. In a study of combined anemia and severe trauma, 198 patients were observed, with 71% of them having penetrating injuries [21]. In a retrospective analysis at 10 minutes after the hospital admission, these trauma patients were divided into 4 groups: hematocrit levels of >40%, 37% to 40%, 33% to 36%, and <33%. The lowest hematocrit groups had the highest incidence of hypotension, acidosis, cognitive disturbances, most extensive injuries, most blood loss, and need of vasoactive medications and transfusions. These observations have also been confirmed in large cohorts of trauma patients, including head trauma, and emphasize the role of anemia as a risk predictor [22]. However, a restrictive transfusion policy is generally recommended [23,24]. Severely injured patients will compensate with, eg, tissue factor activity and thrombin generation due to blood interacting with various broadly damaged tissue parts, including bone in the case of fractures.

Another example in addition to trauma surgery is cardiac surgery. Again, here, as in the patients with atrial fibrillation subjected to long-term anticoagulation medication, the baseline hemoglobin levels predicted how the patients tolerated suddenly worsening anemia during surgery. A drop of 50% of hemoglobin from the baseline level was critical in a large study covering more than 10,179 cardiac patients [25]. The degree of RBC decrease during surgery, often associated with large volumes of blood loss, and need of reoperation due to hemostasis problems led to a HR of 1.53 for in-hospital death, stroke, or kidney failure.

**FIGURE 3:**
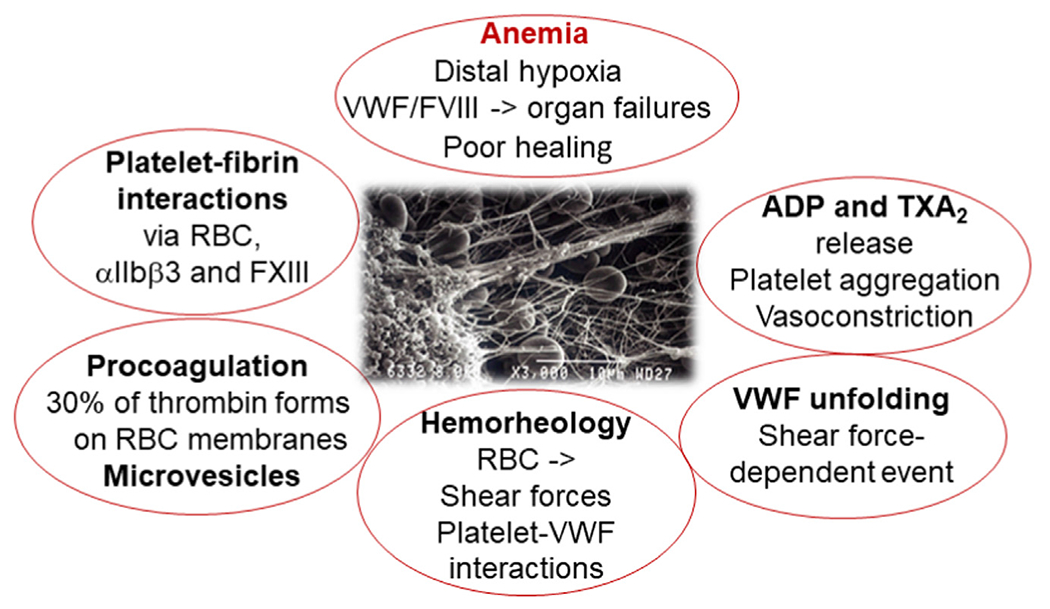
Essential roles of red blood cells (RBCs) in vascular interactions in hemostasis. ADP, adenosine diphosphate; FVIII, factor VIII; FXIII, factor XIII; TXA_2_, thromboxane A_2_; VWF, von Willebrand factor.

**FIGURE 4:**
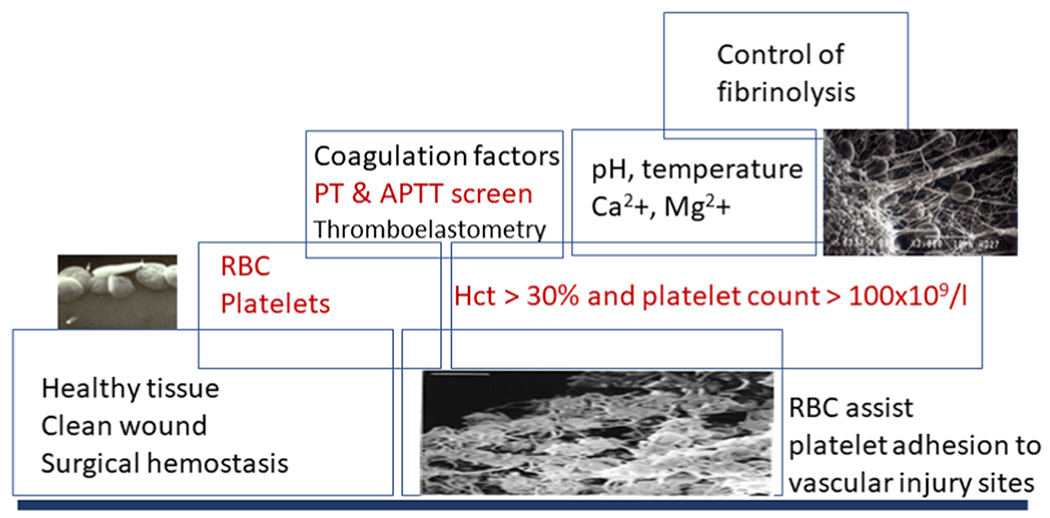
The building blocks of hemostasis important to be recognized and corrected in anemia are indicated in boxes. Overlapping of boxes refers to overlapping mechanisms. Tissue injury and vascular wounds need to be healed by all various aspects of blood clotting, including red blood cells (RBCs), platelets, coagulation factors, and physiological cations. The concentrations of RBCs (hematocrit [Hct]) and platelets are basic elements to initiate clotting. Diagnostic laboratory tests for initial screening of coagulation capacity consist of the prothrombin time (PT) and activated partial thromboplastin time (APTT). The cellular components and mechanical strength of the clot can be evaluated with thromboelastometry. Normal cationic levels, body temperature, and pH assist hemostasis by overlapping with the cellular and coagulation activities. Control of fibrinolysis to avoid bleeding can be supported by the inhibitor tranexamic acid when needed. In case of major bleeds, demanding also surgical interventions, the best outcomes can be achieved by simultaneously supporting all these building blocks. Postoperative thromboprophylaxis needs to be commenced according to the thrombotic risk factors of the patient and the surgical procedure.

For these procedures of surgery for solid tissue support, management should include careful laboratory follow-up to provide appropriate cell and factor replacements, thereby avoiding triggers for unnecessary risk of thrombosis. For the patients with inherently deficient hemostasis who may face life-threatening bleeds, the evaluation of RBC transfusion should be simultaneous with assessment of the coagulation system. This consideration is critical also in case of breakthrough bleeds and success of replacement therapy in patients with hemophilia [6]. The general topics to be monitored in the laboratory include the success of coagulation factor replacement, support of platelet transfusion and temporal discontinuation and/or recuperation from the antithrombotic medication with an antidote, and the control of fibrinolysis to achieve the best outcome in cases of highly vulnerable patients (Figures 3 and 4).

#### 2.2.3 | Organ failure

Anemia is typical in heart failure as well as renal and liver impairment partially due to similar mechanisms involving the inflammation associated with chronic disease. Hepcidin is elevated, which can cause iron dysregulation with hypoferremia and lead to anemia, frequent in inflammatory diseases [26]. Heart and renal failure are typical conditions after cardiac surgery or trauma surgery in the elderly population. Anemia leads to the worsening of organ failure, as hypoxia results in enhanced release of FVIII and VWF [27] (Figure 3). In heart failure, recent recommendations support active management of iron deficiency anemia [28], but debate is ongoing concerning the best diagnostic laboratory tools and criteria. In addition, the interference of inflammation provides challenges to the practical management of these patients. In chronic kidney disease, both erythropoietin synthesis and iron metabolism are impaired, leading to renal anemia, which impacts half of the patients [29]. The aim of managing anemia in renal patients is to adjust hemoglobin levels as close to normal as possible, usually after iron- or erythropoiesis-stimulating agents and vitamin supplementation.

Liver-impaired patients have achieved a new balance of coagulation as their vitamin K–dependent pro- and anticoagulant proteins (proteins C and S) are both reduced, but their FVIII and VWF levels are high due to increased endothelial release and impaired elimination [30]. As a result, prevention of bleeding should not be aimed at correcting the results of conventional coagulation assays, but treatment of anemia may be considered. An increase in VWF and size of VWF multimers has also been reported in sickle cell disease [31], which needs to be considered during transfusions in these patients.

In association with organ failures outside an intensive care setting, the comprehensive analysis of clinical risk factors for bleeds and thrombosis and of laboratory values, including inflammation biomarkers, ferritin, transferrin saturation, and a coagulation screen, will provide an educated basis to find the best possible treatment and follow-up choices. Usually, successful treatment of inflammation tends to correct hepcidin, iron metabolism, and therein anemia, without any other interventions if they are not necessary.

#### 2.2.4 | Pregnancy, delivery, and postpartum hemorrhage

In general, anemia is associated with poor outcomes during pregnancy and at delivery and especially in women with VWD or other hemostatic defects [32,33]. Therefore, anemia should be proactively diagnosed and managed early in pregnancy. Again, since we know that VWD and other inherited defects, including the carriers of hemophilia, have a risk of severe hemorrhage, even during the longer postpartum period, a proactive policy to manage anemia will be important. Pregnant woman and the growing fetus will consume a lot of iron, and the daily demand almost doubles in comparison with a nonpregnant woman. As normal delivery is associated with uterine bleeds, the appropriate iron stores are needed to compensate for the potential blood losses. Local practical guidance to be prepared for and manage acute postpartum hemorrhage is highly recommended [34].

#### 2.2.5 | Recurrent urogenital and GI bleeds

Heavy menstrual bleeds have been recognized as a significant problem in women with VWD, hemophilia carriers, and women with allied bleeding disorders. The heavy, once-a-month or even more frequently occurring bleeding tendency may also underlie pregnancy-related problems in terms of fertility, and development of the aforementioned adverse events during pregnancy, at delivery, and postpartum [12,32,34]. The most obvious and manageable condition of iron deficiency should be in focus regarding heavy menstrual bleeds. Anemia leads not only to impaired quality of life but also to poor control of the bleeds and healing of the bleeding sites. This applies to all other primary, often mucocutaneous, sites of the urogenital and GI tracts. These bleeds, compromising hemostasis, tend to reoccur and therein lead to loss and decreased production of RBCs if iron storage is deficient.

Hematuria is often associated with antithrombotic therapy, cancer, and urological operations [18,24,29]. Although hematuria is usually considered as a nonmajor bleed, it may become difficult to manage and typically reoccur. One underlying cause to be excluded, in addition to local tissue injuries, interventions, and infections, is iron deficiency further compromising the hemostatic capacity. The literature on this topic is scant and often relates to small case studies of glomerular or nephropathic diseases and sickle cell or hemolytic conditions, which are beyond the scope of this review.

The knowledge about recognition, diagnosis, and management of iron deficiency continues to be underrated in clinical practice. Instead, the diagnostic endoscopic criteria for acute and occult bleeds seem clear [35], but hemostatic issues may be out of the focus of the managing clinicians.

In acute settings, the patient blood management program focuses on the avoidance of unnecessary blood transfusions [23]. The highest RBC transfusion targets have been advocated for patients having cardiovascular disease, especially in acute forms, ischemia, and massive bleeds. The program wisely urges the prevention of anemia, but, if prevention has not occurred, transfusions provide an acute solution. In addition, these acute blood transfusion guidelines lack specific guidance on inherited bleeding disorders, which carry a permanent risk for bleeds at low factor levels. Therein, the conservative RBC transfusion limits do not necessarily apply, and the correction of coagulation factor levels alone is not enough, as hemostasis relies on synergistic actions of several mechanisms.

To sum up, in clinics, whatever the nature of the bleeding disorder, early primary hemostasis impairment by reduced RBC numbers and the presence of anemia are to be avoided if possible. The cause of anemia needs to be clarified and managed accordingly, with iron deficiency most often being the hematological background. The active evaluation of RBCs and implementation of specific laboratory diagnosis for anemia should be considered a priority rather than an afterthought in the follow-up of patients with hemostasis and thrombosis issues. To close the circle, this review continues to further discuss what clinicians can learn from basic science and some of the latest observations that can be translated into patient management, whenever possible.

## 3 | MECHANISMS AND BEYOND

**FIGURE 5:**
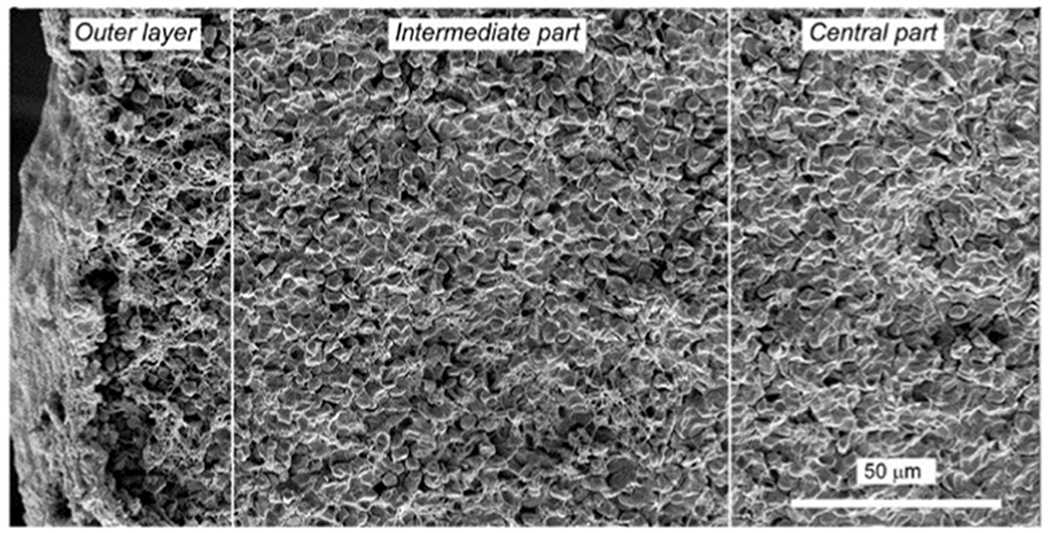
Spatial redistribution of blood clot components during contraction. Panoramic scanning electron micrograph of a contracted blood clot showing: outer layer with much fibrin and aggregated platelets and a few nondeformed RBCs; intermediate part with a mixture of fully and partially deformed RBCs with some intercellular spaces and fibrin fibers, and central part with tightly packed, tessellated polyhedrocytes without spaces and no fibrin. *Source:* Reproduced with permission from Litvinov and Weisel [39].

The clinical entities presented share multiple general mechanisms by which RBCs impact hemostasis (Figure 2, 3 and 5). First of all, RBCs represent the largest numbers of blood cells (at approximately 4 × 10^12^/L vs 3 × 10^11^/L [platelets] and 4 × 10^9^/L [leukocytes]). Since RBCs provide their mechanical force on platelets, they safeguard hemostasis, enhancing the immediate chance for platelets to adhere to the vessel wall upon any injuries and serve to cease a bleed. The improved platelet margination and enhanced viscosity by increasing RBC numbers will also improve the hemostatic properties of VWF, exposing its multiple binding sites for platelets [4,36]. One may sometimes wonder why transfusion of 1 unit of RBCs is able to cease a bleed. It has been demonstrated *in vitro* that RBCs are a significant source of phosphatidylserine, which provides a surface for the generation of thrombin. Accordingly, RBCs increase thrombin generation and consequently platelet activation, as well as fibrin formation and clot stability [1,4]. The more platelet activation and release of regulators of fibrinolysis and the more thrombin generation and fibrin formed, the less local fibrinolysis ensues, thereby stabilizing hemostasis.

Locally, RBCs also secrete vasoconstrictive agents, such as adenosine diphosphate and thromboxane A_2_, not only enhancing local shear rates but also activating and aggregating platelets [36,37]. Furthermore, the procoagulant RBC membranes have a capacity to generate about 30% of the thrombin that whole blood does provide [4].

## 4 | BLOOD CLOT CONTRACTION

Clot contraction or retraction is the shrinkage of the volume of a clot, which has been a mostly neglected and understudied aspect of hemostasis, although it has been thought to be important for making a better seal to stem bleeding, to reduce the obstructiveness of clots, and to aid in wound healing. Therefore, clot contraction is likely important for several aspects of hemostasis in anemia, but much remains unknown, especially with *in vivo* studies lacking.

Once clotting begins, platelets pulling on fibrin via interactions with integrin αIIbβ3 contract the clot, trapping RBCs on the interior, while platelets and fibrin redistribute to the exterior [6,7,38,39]. The cellular mechanism of clot contraction involves platelet filopodia pulling on fibrin fibers, causing a kink in the fiber and accumulation of fibrin around the platelets [40]. Inside the platelet, nonmuscle myosin IIA pulls on actin, which is attached to the membrane integrin αIIbβ3 via talin, vinculin, and other adapter proteins [39]. People with mutations in the *MYH9* gene for nonmuscle myosin IIA often suffer from bleeding problems, but attributing that outcome to clot contraction has been difficult because this defect usually gives rise to macrothrombocytopenia. Recently, it has been shown that some patients with MYH9-related disease who have normal platelet volume still have significant bleeding, suggesting that the bleeding really is a consequence of the loss of clot contraction [41–43]. In addition, studies of individual platelets from patients with *MYH9* mutations have shown that the force exerted by these platelets is lower than that in controls [41,44,45].

Variable numbers of RBCs are trapped in a clot or thrombus, with larger amounts present in venous clots or thrombi, mainly as a result of lower shear rates. Factor XIIIa crosslinking of fibrin α chains is also important for the retention of RBCs in contracted clots, especially in venous thrombi [5]. Although the presence of RBCs in clots was thought to arise merely from them being passively trapped and this is certainly the case, it has been shown that fibrin(ogen) can also act as a specific bridge between RBCs, probably via binding to a β3 integrin on RBCs [46]. During contraction, RBCs accumulate on the interior of the clot, whereas platelets and fibrin are present mainly on the exterior (Figure 5), which is a consequence of the mechanosensing function of platelets to prefer a stiffer substrate [45].

More RBCs are present in venous clots or thrombi, but surprising numbers are present in arterial thrombi [47]. The spatiotemporal distribution of components in coronary artery thrombi, containing mainly platelets at the site of initiation and RBCs mainly at the other end, may also apply to hemostatic clots [48]. The RBCs in the interior of clots are compressed by the force of contraction to a polyhedral shape or forms intermediate between biconcave and polyhedral [7,49]. As a result, the deformed cells, named polyhedrocytes or piezocytes, form a tessellated array that makes a nearly impermeable seal (Figure 5), which appears to be important for hemostasis.

In a mouse model of hemophilia A with longer bleeding times, clots contained a smaller proportion of compressed polyhedrocytes or intermediate forms, making the clots more permeable [6]. Clot contraction also affects both fibrinolysis and therapeutic thrombolysis [50]. According to *in vitro* studies, internal fibrinolysis (model for physiological fibrinolysis) is faster in contracted clots, whereas external fibrinolysis (model for therapeutic thrombolysis) is slower in contracted clots. Clot contraction stiffens clots, making them mechanically stronger and more resistant to external forces [51]. Moreover, since platelets are mechanosensing cells, they pull harder against a stiffer substrate, which means that platelets exert more force in a clot with more RBCs. Consequently, *in vitro* experiments have shown that platelets exert more force in clots made from blood with higher hematocrit [51].

The implication is that with higher hematocrit, clots will contain more RBCs, platelets will exert more force, and the clot will make a better seal. Therefore, another result of low hematocrit *in vivo* could be less contracted clots that are not as effective to stem bleeding, although this remains to be demonstrated, since *in vivo* clot structure is strongly dependent on blood flow [3].

## 5 | MICROVESICLES

Microvesicles of platelets and of RBC origin are better known for enhancing thrombosis, but they contribute to hemostasis since they represent a means for intercellular communication [52]. With smaller RBC numbers, fewer microvesicles might be released. Under hemolytic (eg, paroxysmal nocturnal hemoglobinuria) or sickling conditions, adenosine diphosphate and consumption of nitric oxide may lead to thrombotic complications, including release of microvesicles [53]. This is also relevant for immunothrombosis, ie, antiphospholipid antibody syndrome, and thrombotic thrombocytopenic purpura, where thrombin generation is enhanced and platelet-VWF interaction is overactive due to ADAMTS-13 deficiency [53]. Under these conditions, platelets and RBCs may shed microvesicles, which support hemostasis, but can also be thrombogenic.

The formation of microvesicles follows the loss of membrane asymmetry and presence of phosphatidylserine on their surface [52]. Also, the storage lesion of RBCs may lead to microvesicle release, which is one mechanism responsible for the harmful effects of RBC transfusion, including an increased incidence of deep vein thrombosis and other prothrombotic conditions [54]. Higher levels of hypercoagulable microvesicles in the blood are associated with increased generation of thrombin and reduction of clotting time, mostly from expression of phosphatidylserine [55]. The microvesicles in the circulation can also internalize heme and transfer it to the endothelium, activating it and initiating thrombosis. Microvesicles can also bind fibrin and maintain the procoagulant phenotype for a prolonged time [56]. All these effects are relevant to anemia, impacting the balance between thrombosis and hemostasis.

## 6 | CONCLUSIONS

Although there has long been observational evidence that RBCs play a role in hemostasis, it is only recently that some of the mechanisms have been discovered. Nevertheless, this remains a rich area for both basic and clinical studies since much is still unknown.

Particularly, a comprehensive analysis of sets of both clinical and laboratory-based variables as risk factors for bleeding and thrombosis should be taken into account. A clean wound and exposed collagen surface, enough platelets and RBCs with their functional capacities, good coagulation factor activity and cationic environment, and appropriate fibrinolysis are the building blocks of hemostasis (Figure 4). Educational efforts related to how to interpret the currently available clinical laboratory testing demand constant attention. Unfortunately, the current transfusion guidelines do not provide guidance on special groups of patients, namely, severe inherited and acquired bleeding disorders. We will need more practical tools to reach goals for the efficacious and safe management of our patients with anemia and coagulation disorders.

## ABBREVIATIONS

RBC: red blood cells
VWD: von Willebrand disease
VWF: von Willebrand factor
